# Heart and bladder detection and segmentation on FDG PET/CT by deep learning

**DOI:** 10.1186/s12880-022-00785-7

**Published:** 2022-03-30

**Authors:** Xiaoyong Wang, Skander Jemaa, Jill Fredrickson, Alexandre Fernandez Coimbra, Tina Nielsen, Alex De Crespigny, Thomas Bengtsson, Richard A. D. Carano

**Affiliations:** 1 Genentech, Inc., South San Francisco, CA USA; 2 F. Hoffman-La Roche Ltd., Basel, Switzerland

**Keywords:** FDG PET/CT, Segmentation, Physiological noise, Deep learning

## Abstract

**Purpose:**

Positron emission tomography (PET)/ computed tomography (CT) has been extensively used to quantify metabolically active tumors in various oncology indications. However, FDG-PET/CT often encounters false positives in tumor detection due to ^18^fluorodeoxyglucose (FDG) accumulation from the heart and bladder that often exhibit similar FDG uptake as tumors. Thus, it is necessary to eliminate this source of physiological noise. Major challenges for this task include: (1) large inter-patient variability in the appearance for the heart and bladder. (2) The size and shape of bladder or heart may appear different on PET and CT. (3) Tumors can be very close or connected to the heart or bladder.

**Approach:**

A deep learning based approach is proposed to segment the heart and bladder on whole body PET/CT automatically. Two 3D U-Nets were developed separately to segment the heart and bladder, where each network receives the PET and CT as a multi-modal input. Data sets were obtained from retrospective clinical trials and include 575 PET/CT for heart segmentation and 538 for bladder segmentation.

**Results:**

The models were evaluated on a test set from an independent trial and achieved a Dice Similarity Coefficient (DSC) of 0.96 for heart segmentation and 0.95 for bladder segmentation, Average Surface Distance (ASD) of 0.44 mm on heart and 0.90 mm on bladder.

**Conclusions:**

This methodology could be a valuable component to the FDG-PET/CT data processing chain by removing FDG physiological noise associated with heart and/or bladder accumulation prior to image analysis by manual, semi- or automated tumor analysis methods.

## Introduction

Whole body positron emission tomography (PET)/computed tomography (CT) with ^18^fluorodeoxyglucose (FDG) has been widely used in diagnosis, staging and re-staging for various cancers [1–5]. Baseline total metabolic tumor volume (TMTV) has been found to be prognostic, where TMTV correlates significantly with overall survival (OS); in addition, the change in TMTV for follow-up scans can be used to assess an early treatment response [6–9]. Although PET is very sensitive to detect tumors with high metabolic activity, uptakes of FDG is not specific to tumors. FDG accumulation can occur at areas of inflammation or infection, in normal tissues with high metabolic activity such as brain and working muscles, and FDG can accumulate in excretory/filtration organ systems. As such, this accumulation can appear as false positives for tumor assessment in FDG-PET images [10–13]. The uptake in the bladder and heart is most problematic since it often present and just varies by degree. More specifically, the urinary bladder usually contains a variable amount of contrast agent or radiotracer accumulation post contrast administration and can appear comparable in FDG uptake as tumors in the abdominal/pelvic region. Similarly, the heart is another potential source of false positive signal due to high blood pool signal and/or local FDG accumulation in heart muscle. If the bladder or heart uptake is identified as tumor by mistake, it can impact the utility of FDG-PET as a prognostic or efficacy assessment, especially in low tumor burden situations at baseline or post treatment.

The automatic image segmentation of FDG-PET images or co-segmentation of FDG-PET/CT images has been extensively studied[14–18]. Almost all of these segmentation approaches have been aimed at lesion or tumor delineation. Region growing[19, 20] and random-walk[21, 22] based methods have also been introduced to utilize spatial information but the performance can suffer when the region of interest does not have good homogeneity. Often these approaches suffer from segmentation leakage to other surrounding objects or background regions. An enhanced random walk method[23, 24] was proposed to solve this issue on head and neck cancer by including k-means clustering to refine target seed and adaptive optimal threshold selection. Active contour algorithm combined with discriminant analysis[25] has been applied to deal with datasets with noise and weak boundaries. Deep learning methods using 2D U-Net[26] was also used to segment tumors on head and neck. It has shown superior performance than other classical machine learning approaches.

There are only a few published studies that describe bladder segmentation methods for FDG-PET/CT imaging data[27, 28]. Gsaxner et al.[27] exploit FDG enhanced PET by simple thresholding to generate ground truth for bladder segmentation on CT. They assumed the bladder on PET and CT are exactly same and there are no tumors in the abdomen. Geoffrey et al.[28] proposed a two-step clustering based method. Two manually-chosen seed points were selected within the bladder and tumor to serve as initial cluster means for K-Means clustering, which was applied to both the PET and CT images simultaneously to segment the bladder. Due to the need for manually selected seed points and a user-specified number of clusters, this method likely lacks the generalizability to be applied to more heterogeneous cases. Fourcade et al.[29] combined superpixels and deep learning approaches to segment organs such as brain, heart and bladder in FDG-PET images from a breast cancer patient. But, they only used FDG-PET image data alone, without CT, and they did not address the difficult situation where the tumors are in close proximity to the organs. Although bladder segmentation has been previously studied, to our knowledge, there is no reported studies regarding heart segmentation for FDG-PET/CT images.

**Fig. 1:**
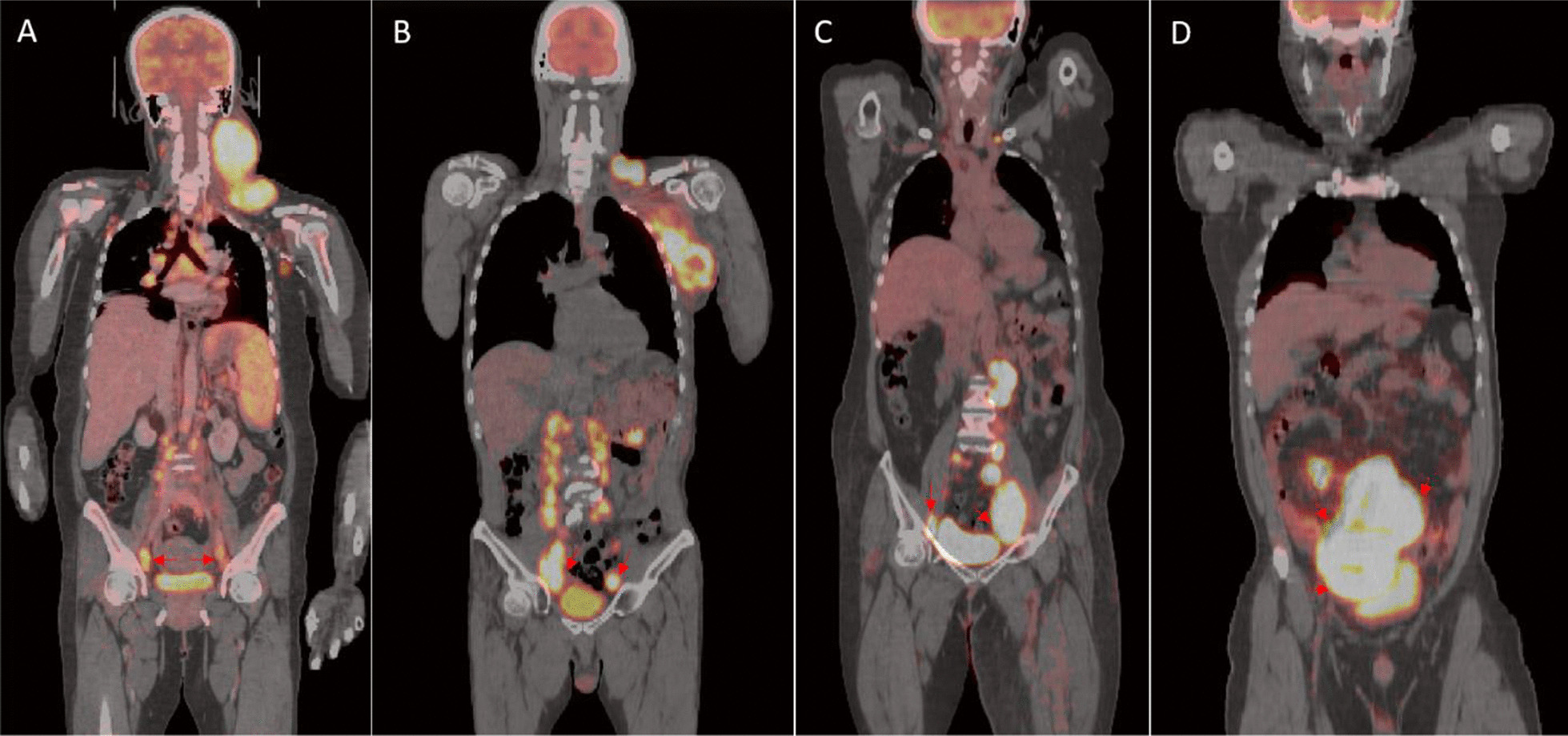
**a, b** Registered PET/CT showing cases where tumors are very close to the bladder. **c, d** Two cases with tumor connected to the bladder. Tumor is pointed out by red arrows

In this study, we proposed a deep learning based method to automatically segment heart and bladder within FDG PET/CT images as a means to suppress this physiological FDG signal from contaminating the use of these scans for tumor characterization or other diagnostic purposes. The challenges in this work includes: (1) there is large inter-patient variability of heart and bladder appearance in these images in terms of intensity, size, shape, position, etc. The automated segmentation approach needs to be generalizable when applied to highly heterogonous cases in practice. (2) FDG PET/CT data are acquired at the same imaging session but not exactly the same time and are generated by different image contrast mechanism. Accordingly, there is no guarantee of one-to-one correspondence [30] meaning the size and shape of bladder or heart can be different on PET and CT scans. Therefore, the segmentation should not be biased towards the CT feature in this setting since our goal is to minimize the FDG signal arising from these organs being erroneously contributing to tumor burden or other diagnostic assessments. (3) Most importantly, there are tumors very close or even connected to the bladder or heart especially in some cases with high tumor burden and it is challenging for conventional computer vision based methods to handle this properly. Figure 1 provides an example where a tumor is in close proximity of the bladder (tumor is indicated by red arrows). The second and third challenges are not fully appreciated and have not been clearly addressed in nuclear imaging literature.

## Materials and methods

### Training and test data

The dataset used in this study was obtained from two clinical trials in Non-Hodgkin Lymphoma (NHL) and both trials received institutional review board (IRB) approval. The training set was from a clinical trial called GOYA (NCT01287741 [31]) and they were randomly selected to include case with low, medium and high treatment burden. In total, 538 scans of co-registered FDG-PET/CT pairs were obtained from 519 patients (256 male, 263 female) with diffuse large B-cell lymphoma (DLBCL) were used in bladder segmentation training and 575 scans from 547 patients (268 male, 279 female) were used for heart segmentation training. To ensure that the heart segmentation model is generalizable and also works on cases without signal in the heart (i.e. the model does not mistake tumor as heart), the training set also includes 35 scans (out of 575) that were negative samples, where FDG signal is not present in the heart. We also attempted to include similar negative samples for bladder segmentation but could not find such cases in our dataset.

The test set was extracted from an independent data set from the clinical trial GO29781 (NCT02500407 [32]) in B-cell NHL. 103 scans of different patients were used to evaluate bladder segmentation and 113 scans for heart segmentation evaluation (10 of them have no visible signal in the heart).

In total, 26 different scanners that differ based on model types from the three major manufacturers (SIEMENS, PHILIPS, GE) were used. The original matrix size of CT is from [512 × 512 × 137] to [512 × 512 × 671], with corresponding resolution in the range of (0.97 mm, 0.97 mm, 1 mm) to (1.37 mm, 1.37 mm, 5 mm). The original matrix size of PET is [128 × 128 × 170] to [200 × 200 × 546], with corresponding resolution between (4 mm, 4 mm, 2 mm) and (5.47 mm, 5.47 mm, 4 mm).

The ground truth segmentation for both training and test sets were generated by intensity thresholding on PET image only and CT image was used for anatomical reference. Manual correction was applied by an image specialist when needed, e.g. there is tumor close or connected to the heart or bladder.

### Pre-processing

FDG-PET and CT scans were registered and resampled (linear interpolation) to the same resolution of 2 × 2 × 2 mm in pre-processing. Additionally, the training set was augmented by 8 times, including translation, rotation and rescaling. The augmented data were randomly generated, where the amount of translation, rotation, and rescaling was restricted to range: translation (0–10% of image width/height to the left or right, anterior or posterior), rotation (an angle between 0–15 degree with respect to the x–y plane), and rescaling (a factor between 0.9–1.1 of original size).

### Image segmentation

Due to computer memory constraints, it is impossible to fit the whole body FDG-PET/CT scan to build a multi-class segmentation model for both heart and bladder. In addition, it is not necessary to provide all anatomical structure from head to feet to segment bladder and heart. As such, two 3D U-Nets[33] were built, one network to segment heart FDG-uptake and one network to segment FDG-uptake in the bladder. Each network was applied separately and the resultant segmentations were combined into a single physiological FDG uptake mask. Based on the orientation of superior-inferior and slice location, automated image cropping or padding was applied to the thoracic region to focus on heart with a consistent size of 256 × 256 × 256. If the original image is larger than 256, then cropping is used to extract the central volume. For volumes small than 256 × 256 × 256, then padding with the edge value of the image was used to fill the remainder of the 3D volume. The same strategy was used on the abdominal region to target the bladder. In this manner, the field of view is sufficient to include reference landmarks, e.g. pelvic bone for bladder, thoracic aorta for heart.

**Fig. 2:**
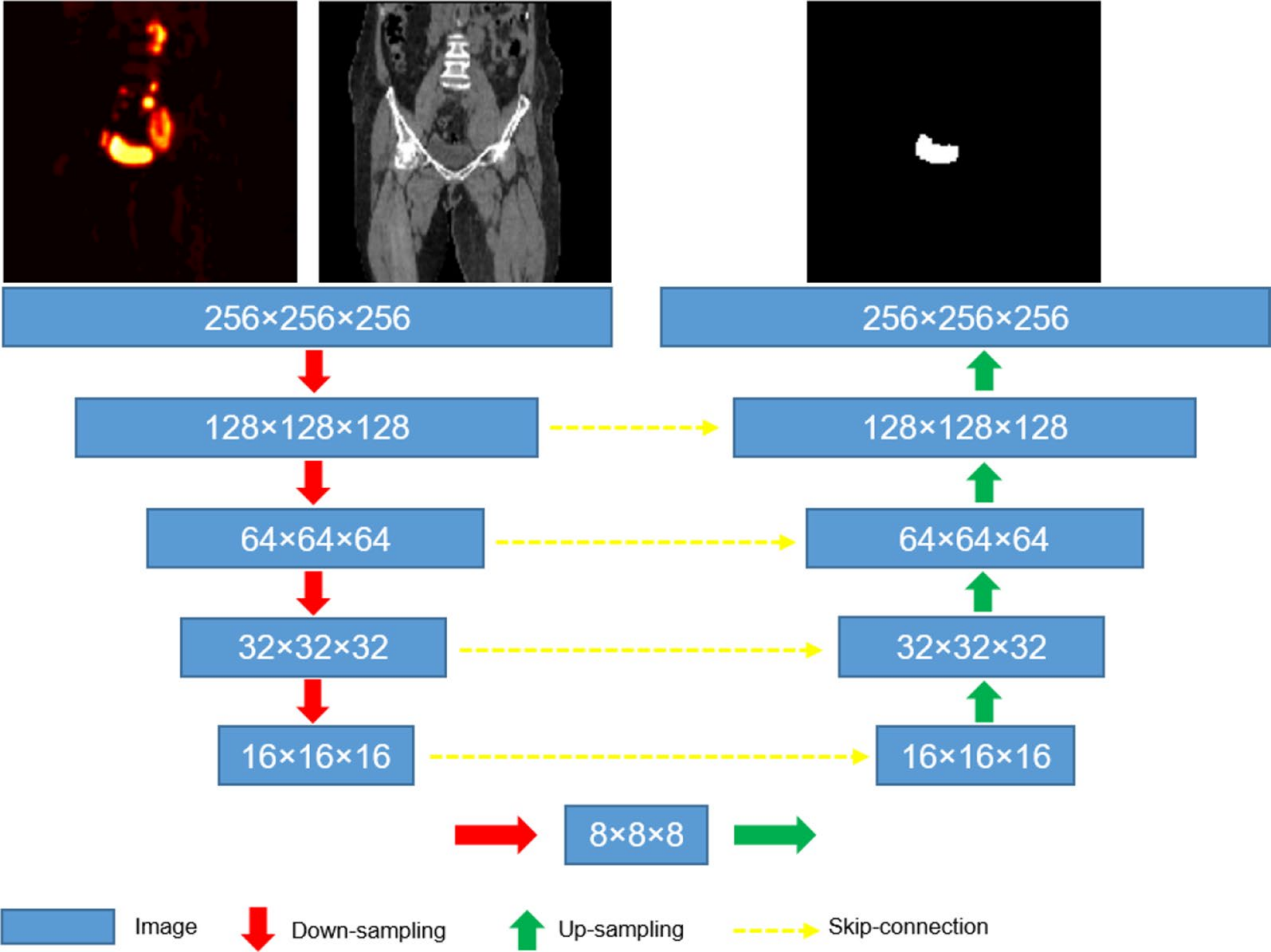
3D U-Net architecture used in bladder and heart segmentation

As shown in Fig. 2, the input to the network is two modalities: one channel for the FDG-PET image data and the other channel for the CT image data and the output is the mask image for the bladder or the heart. Thus, the input size is 8 × 256 × 256 × 256 × 2, where 8 is the batch size. The encoder for the network includes 5 blocks of down-sampling from 256 × 256 × 256 to 8 × 8 × 8 and the decoder has a corresponding 5 up-sampling block to recover the original resolution. Each down-sampling includes 2 convolutional layers and 1 max-pooling layer. Each up-sampling block comprises 2 convolutional layers and 1 layer of 3D up-sampling. Same volumetric kernels of 3 × 3 × 3 were applied in each convolution and skip-connections were used in between to incorporate fine grained details from early layers. Training loss was composed of Dice loss and weighted cross-entropy[34] due to imbalanced classes. The model was trained on multiple NVIDIA Quadro P6000 with 24 GB GPU memory, using an optimizer of Adam with a learning rate of 0.001 and a decay rate of 1e-4.

## Results

The segmentation models were assessed on test sets from an independent clinical trial GO29781 (MOSUN). Specifically, 103 scans (co-registered PET/CT pairs) were used on bladder segmentation and 113 scans on heart segmentation. They were collected from different visits, from baseline scan to a scan at Month 9. Segmentation metrics including Dice Similarity Coefficient (DSC), Hausdorff Distance (HD), and Average Surface Distance (ASD) were used in quantitative evaluation. The bladder segmentation model achieved a mean Dice score of 0.95, HD of 10.3 mm and ASD of 0.9 mm. The heart segmentation model achieved a mean DSC over 0.96, HD of 10.7 mm and ASD of 0.44 mm. Table 1 shows the results of heart segmentation and bladder segmentation on test set by the proposed approach.

**Table 1:** Quantitative evaluation results on heart and bladder segmentation on an independent trial GO29781

	DSC	HD (mm)	ASD (mm)
Bladder segmentation (n = 103)	0.948 ± 0.025	10.292 ± 8.050	0.907 ± 0.447
Heart segmentation (n = 113)	0.966 ± 0.024	10.730 ± 10.71	0.439 ± 0.279

**Fig. 3:**
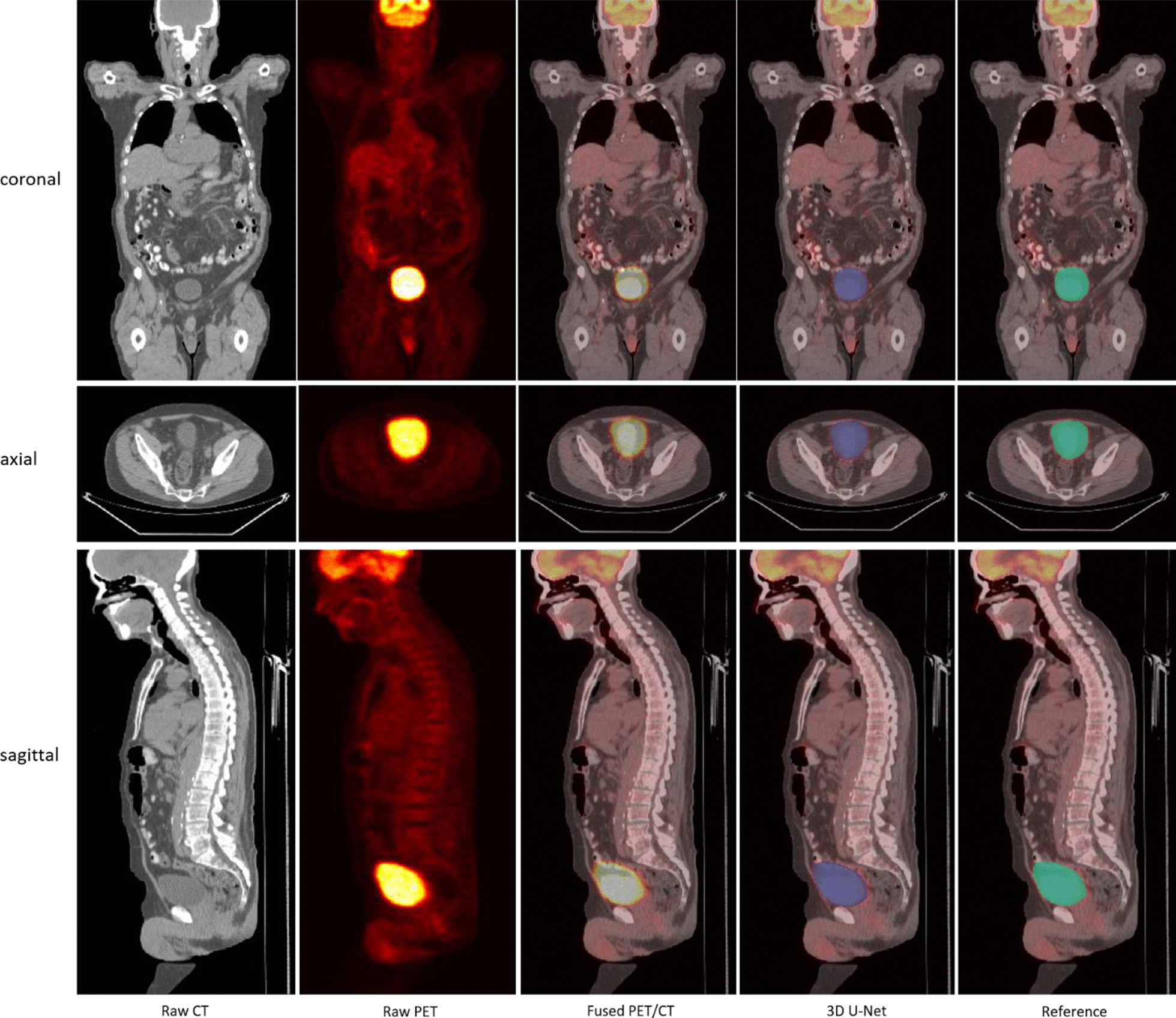
Example of a large bladder and it has a bigger bladder on PET than the one on CT. Automated segmentation is shown in purple and reference segmentation in cyan

Figure 3 shows a segmentation example of large bladder by coronal, axial and sagittal view. The size different can be easily seen between the CT and PET images. The model is able to capture the whole bladder based on the signal from the PET.

**Fig. 4:**
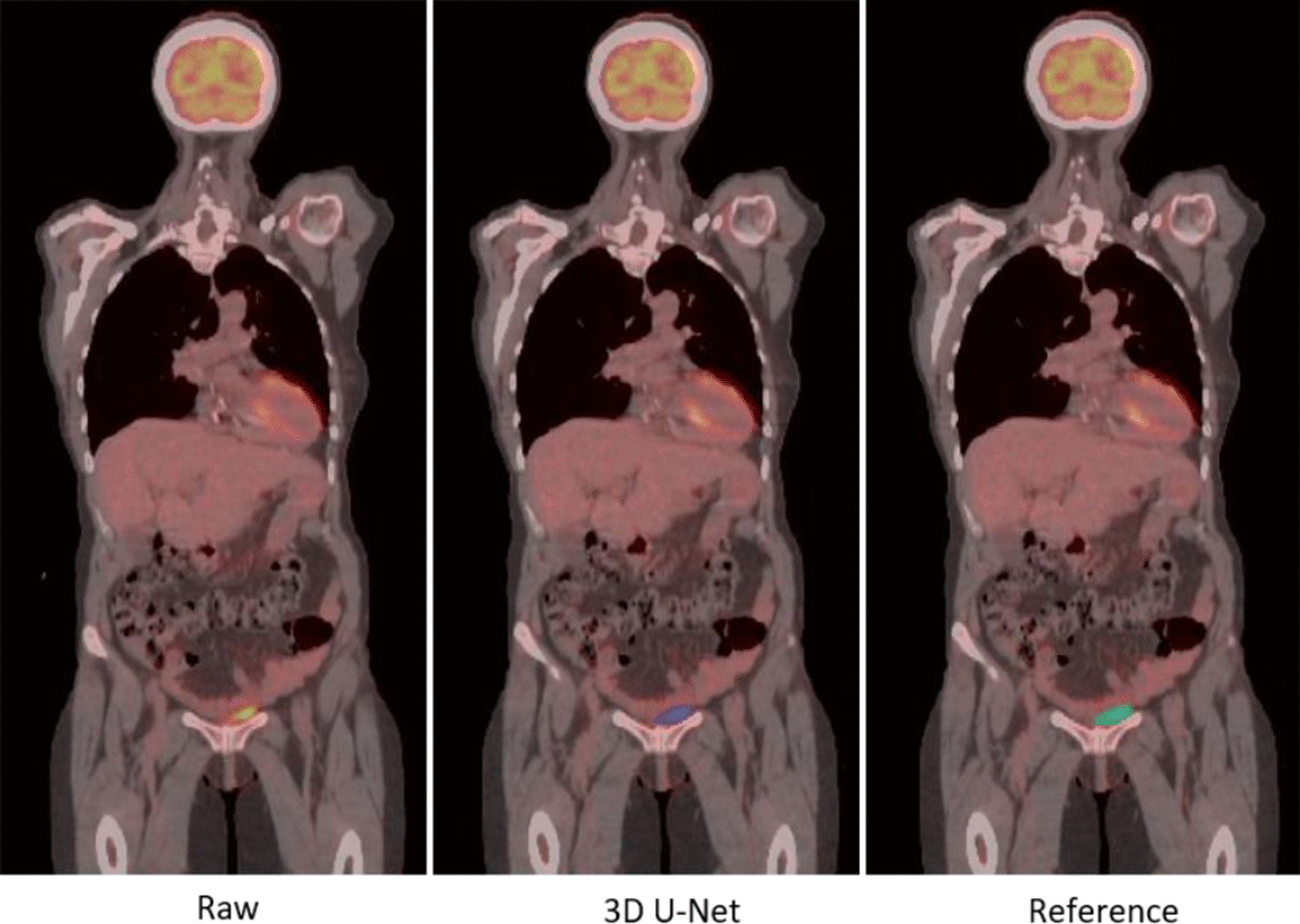
Example of a small bladder in contrast with the large bladder in Fig. 3

**Fig. 5:**
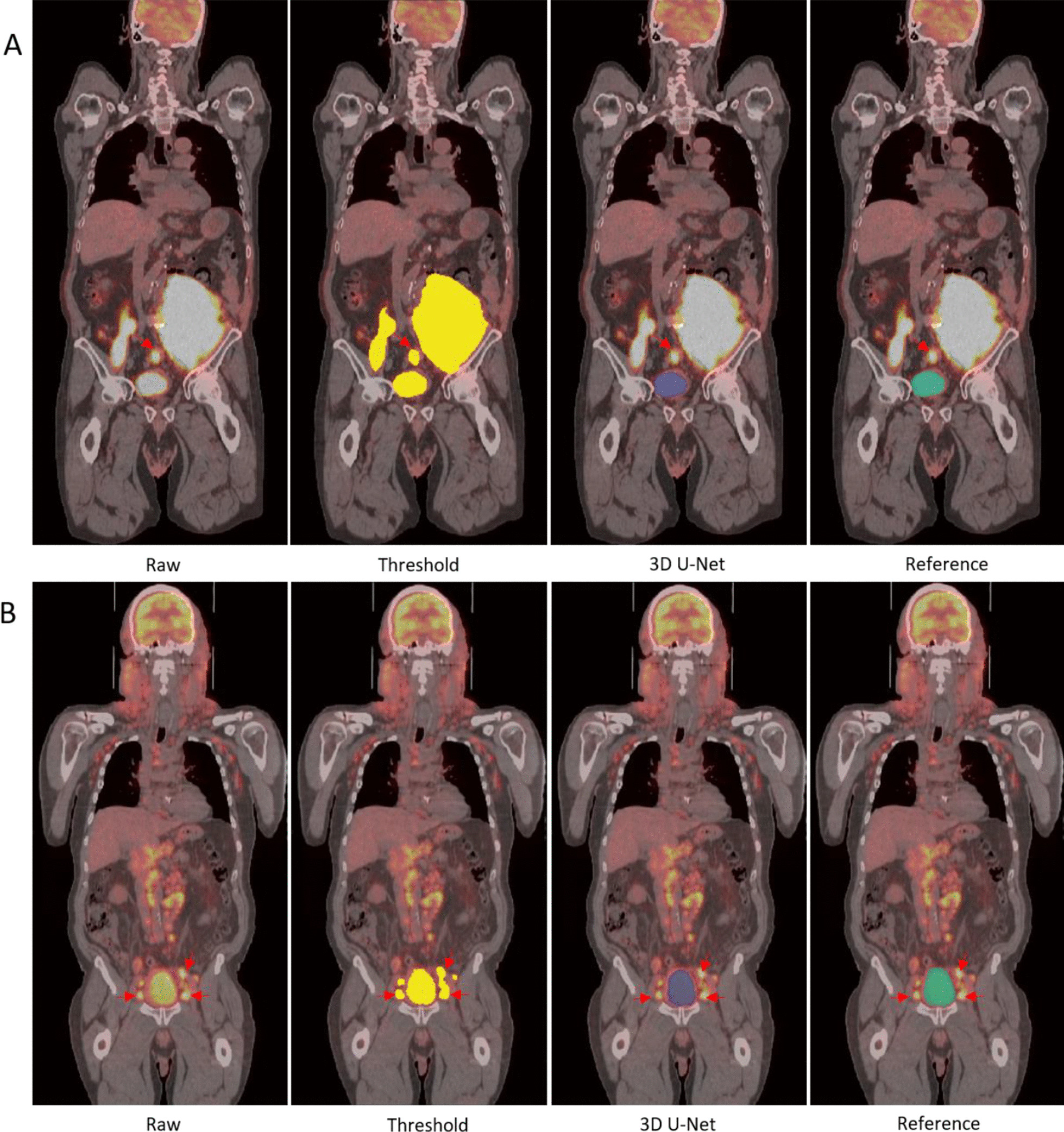
Example of bladder segmentation with tumor nearby indicated by red arrows on PET/CT image. Threshold based segmentation (yellow), automated segmentation (blue) and reference segmentation (green) are present

Figure 4 shows a very small bladder located slightly left of the pelvis. Figure 5 shows two examples with pelvic tumors very close to the bladder. The neighboring tumors are indicated with a red arrow on the original PET-CT image and threshold-based segmentation in the abdomen were also performed for comparison. For case A there is a tumor right above the bladder and two larger ones slightly superior. In case B, there are small tumors on the two lateral sides of the bladder. For both cases, the threshold-based segmentation approached extracted both the tumors and bladder, whereas the 3D U-Net extracted only the bladder.

**Fig. 6:**
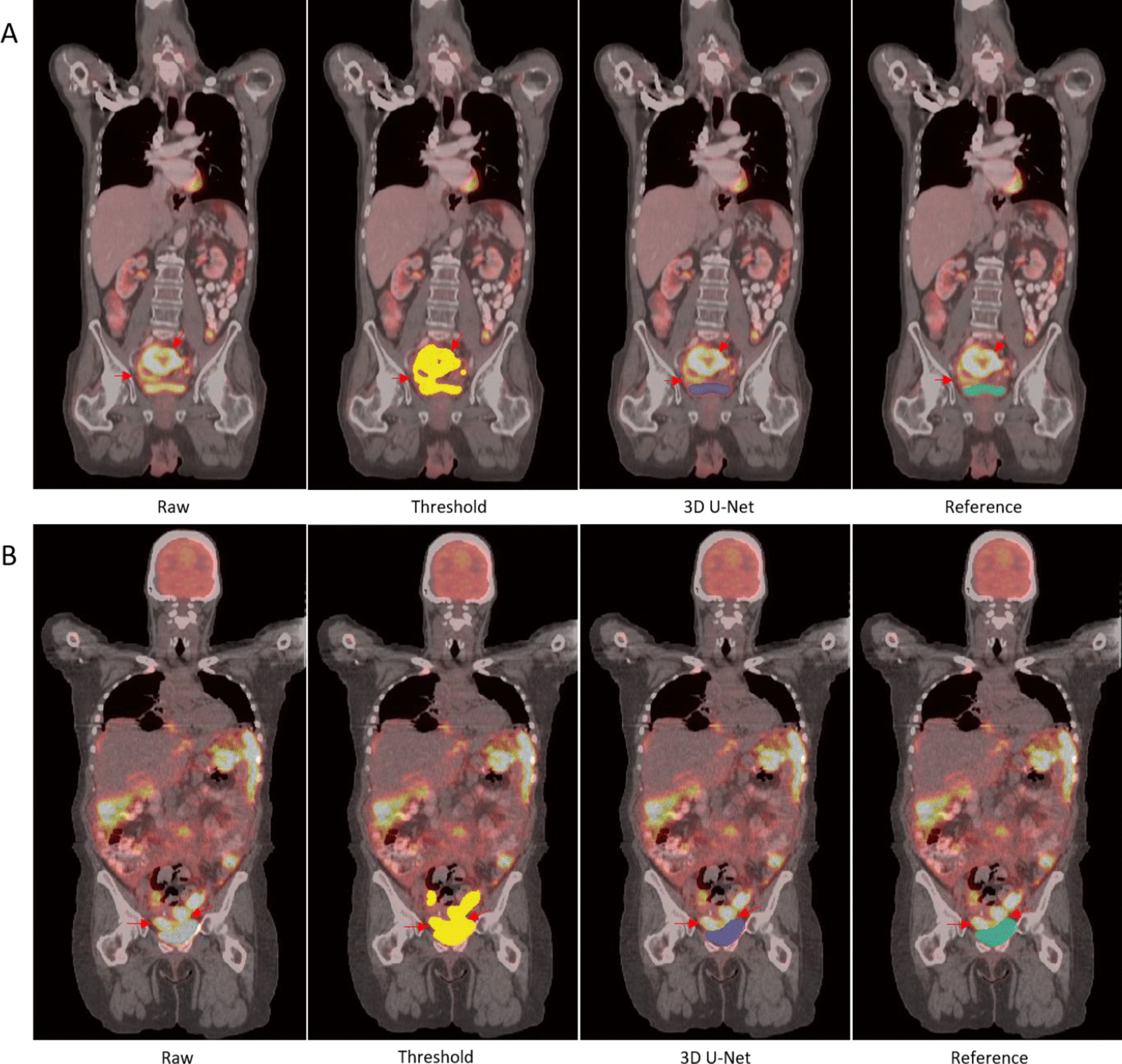
Example of bladder segmentation with tumor attached that is indicated by the red arrow

Furthermore, Fig. 6 presents two cases where pelvic tumors are connected to the bladder. The intensity threshold approach is unable to separate the bladder from the tumor and will require manual intervention to provide the separation. The model succeeded to separate the bladder (blue mask) from the neighboring tumors in both scenarios.

**Fig. 7:**
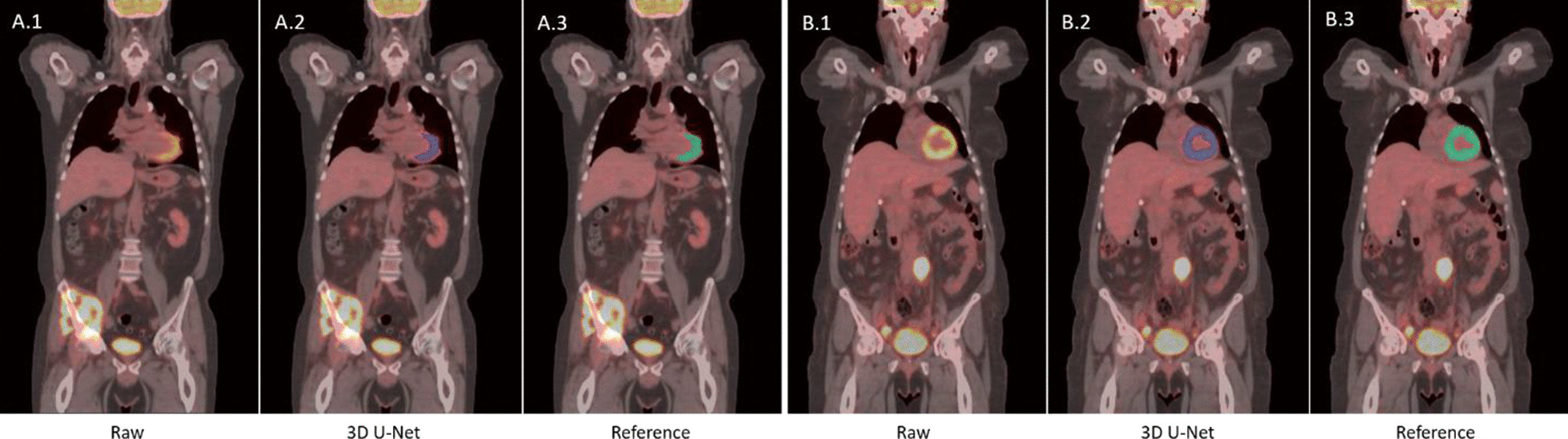
Example of heart segmentation with different degree of FDG uptake in heart: **a** medium uptake and **b** high uptake

Figure 7 provides two examples of heart segmentation with different levels of FDG activity within the heart. The left case (Fig. 7a) has most of the left ventricle filled with tracer, while the right (Fig. 7b) case has high metabolic activity in the majority of heart muscle. The 3D U-Net successfully segmented the heart in both cases.

**Fig. 8:**
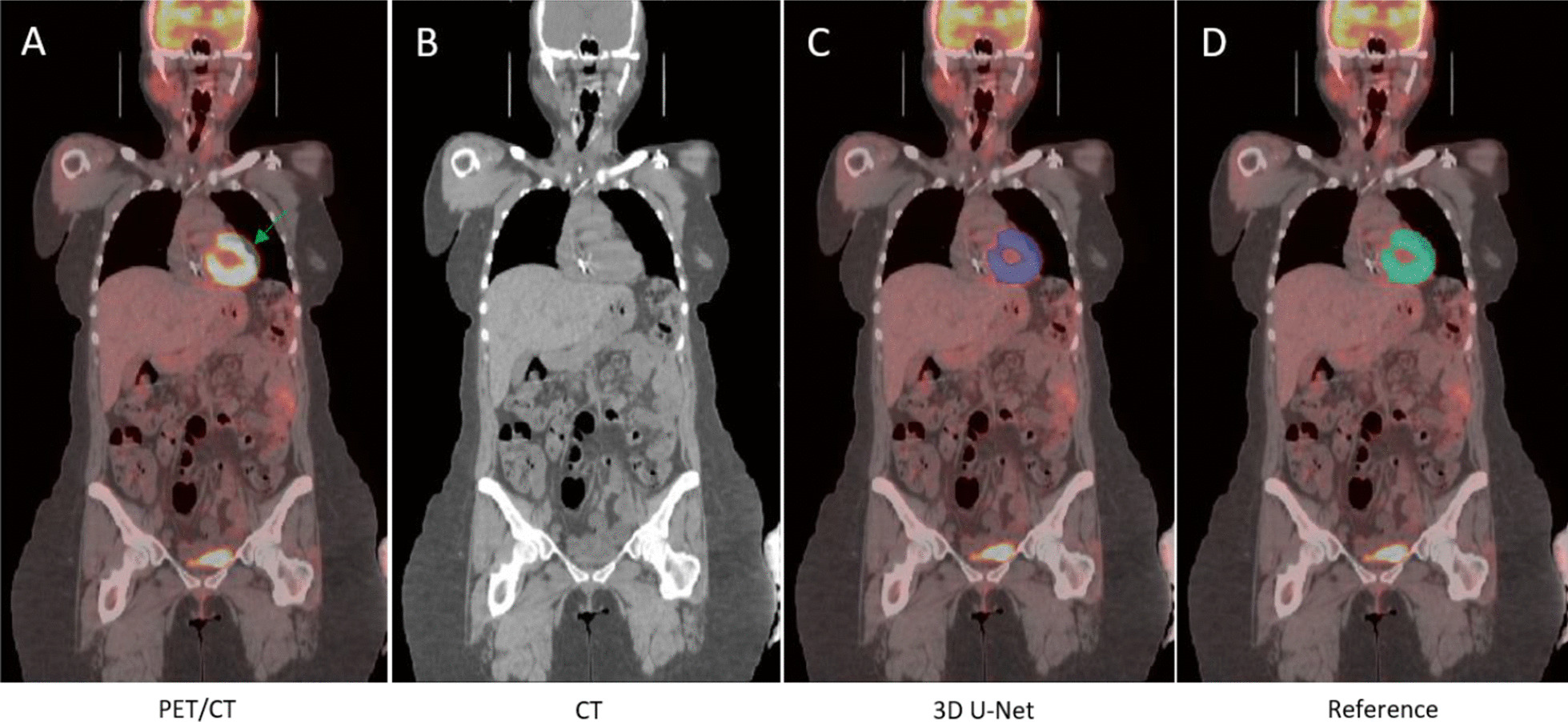
Example of heart segmentation on case where heart appears larger on PET than in the CT. **a** PET-CT, **b** CT, **c** heart segmentation by the model and **d** reference segmentation

Figure 8 contains a case where the apparent heart size is different on PET and CT. The difference can be observed by comparing the co-registered PET/CT (Fig. 8a) and CT (Fig. 8b) image alone. The model succeeded to capture the metabolic region of the heart and is in good agreement with the reference image.

**Fig. 9:**
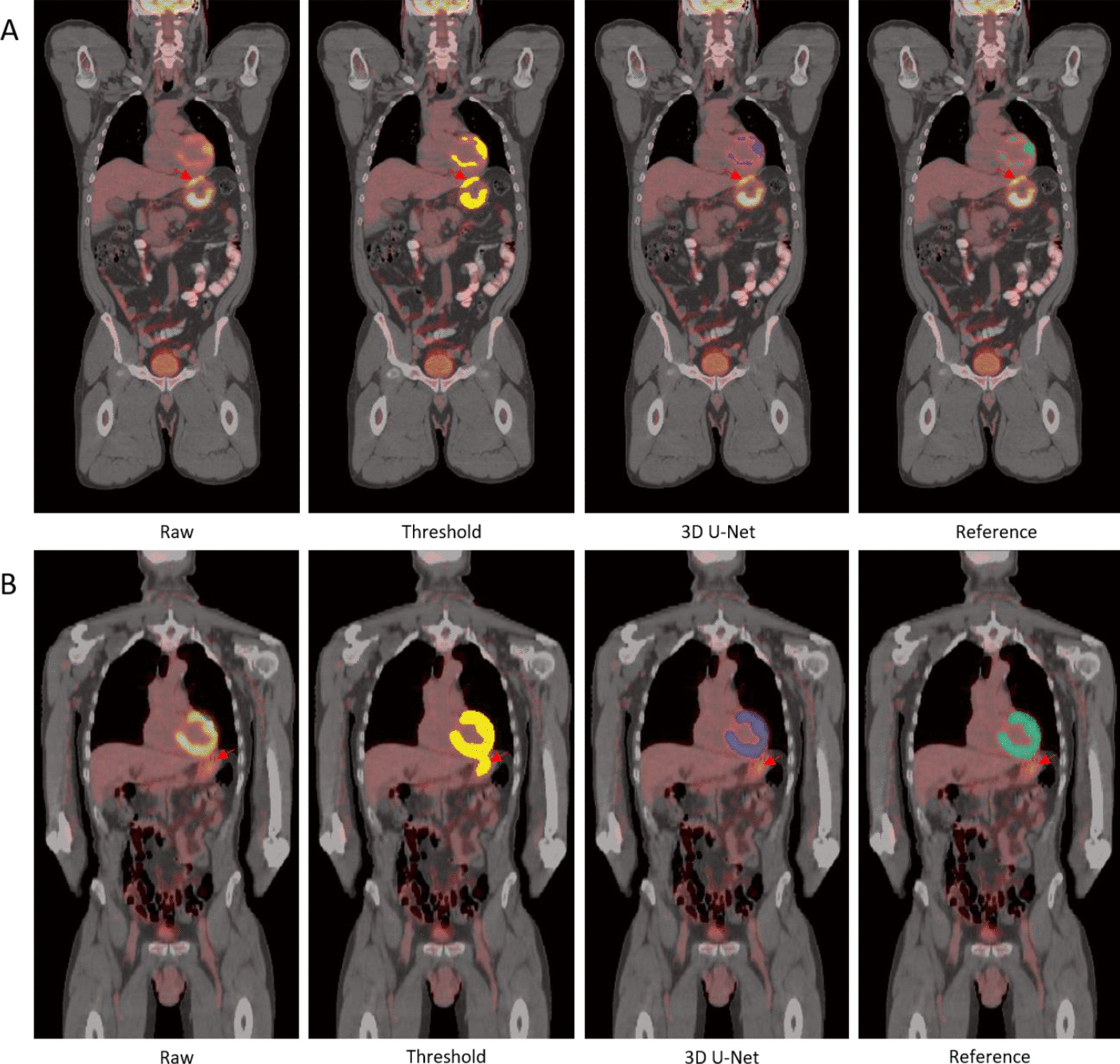
Example of heart segmentation with tumor close by (**a**) and attached (**b**) that is pointed out with red arrow

Two cases with tumors or gastric inflammation close or connected to the heart are shown in Fig. 9. The tumor below the heart is specified by a red arrow. The intensity threshold-based segmentation approach segments both the heart and tumors together and will require manual steps to remove the heart. In both cases, the elevated FDG regions within the heart were correctly segmented by the model.

**Fig. 10:**
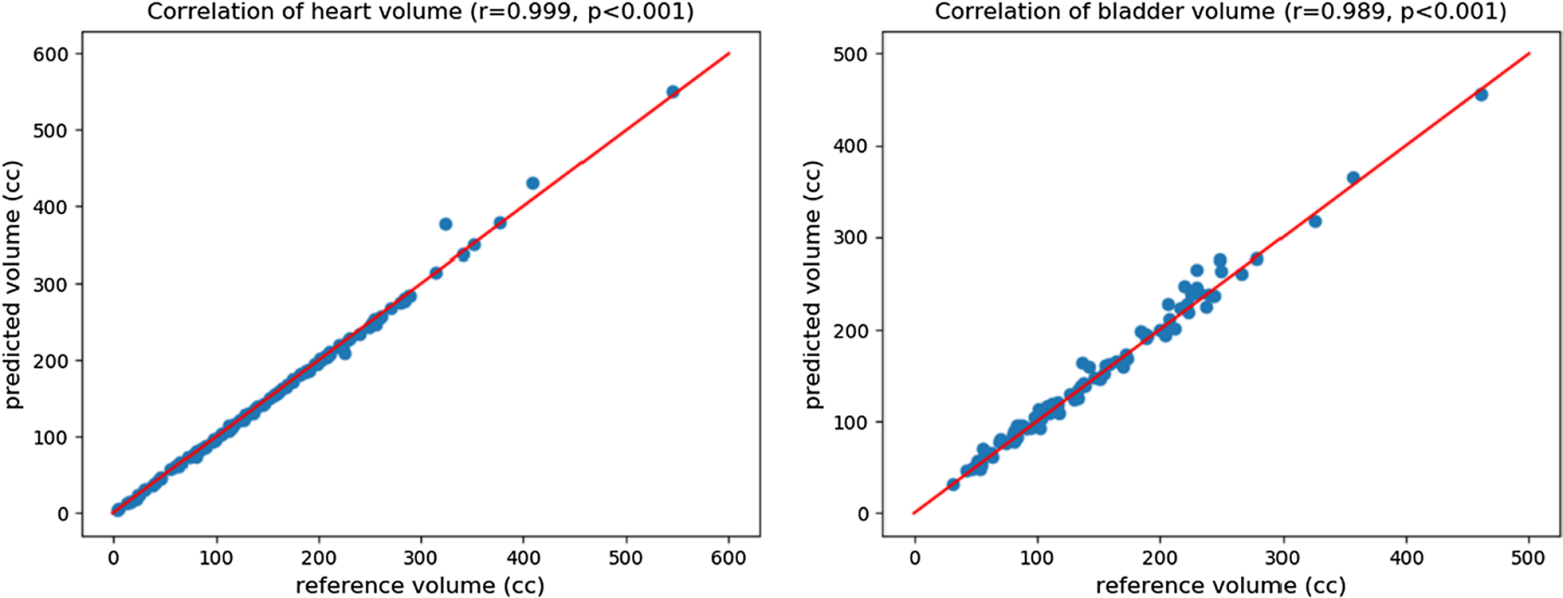
Volume comparison of heart and bladder of automated segmentation and reference

Overall, the heart and bladder segmentation by the 3D-Unet exhibited strong agreement with the ground truth reference estimates. As shown in Fig. 10, the volume of the automatically segmented heart (155.57 ± 96.88 cm^3^) and bladder (148.84 ± 82.66 cm^3^) have a very strong correlation with the reference segmentation (heart: 156.77 ± 95.50 cm^3^, bladder: 145.79 ± 82.32 cm^3^). The spearman’s rank order correlation is 0.999 for heart and 0.989 for bladder respectively.

**Fig. 11:**
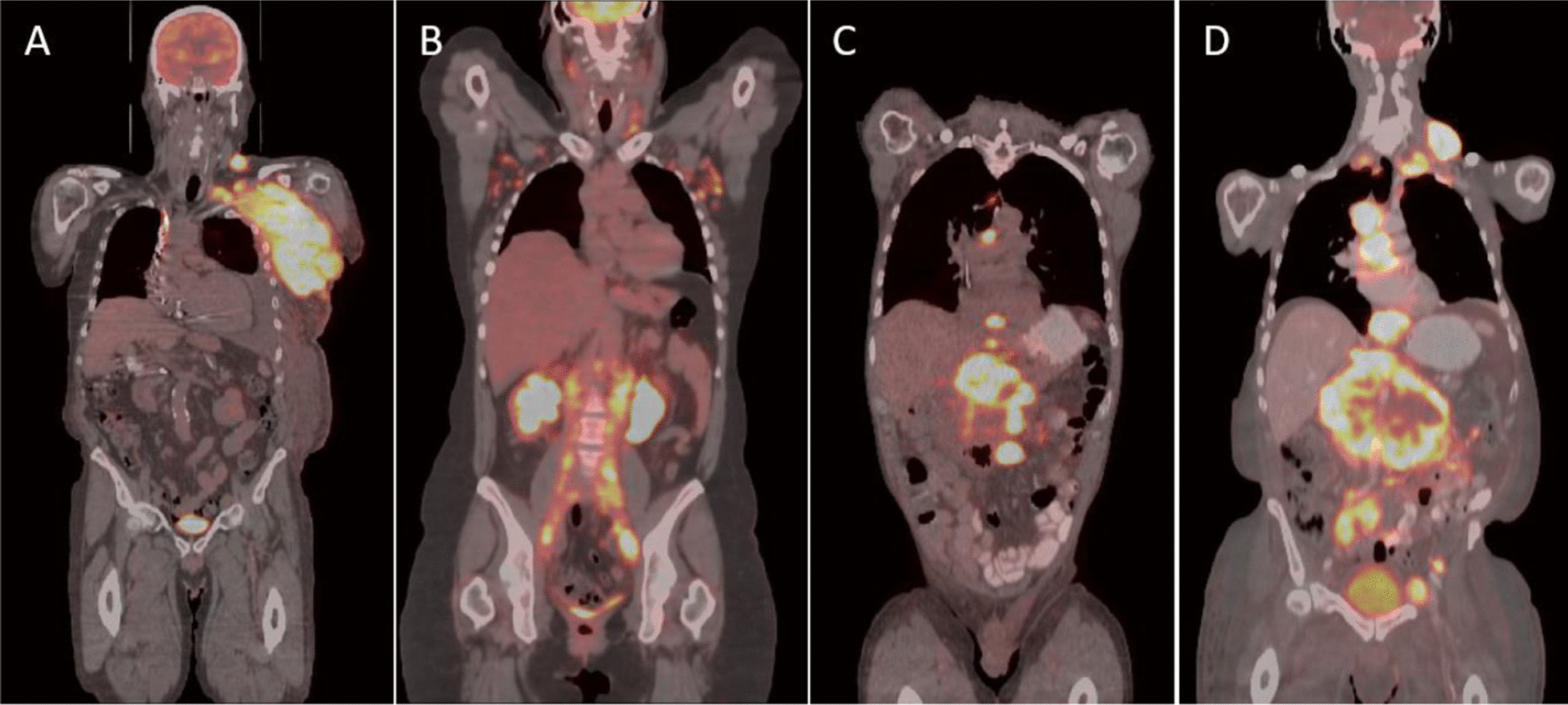
Examples of true negative by the heart segmentation model. There is no FDG uptake in the heart but in the neighboring regions

The heart segmentation test set includes 10 negative samples without FDG uptake in the heart, although positive FDG signal was present in neighboring tissue outside the heart (Fig. 11). As a result, no FDG positive voxels were identified as heart by the model although there is high metabolic tumor in the chest or abdomen. As shown in Table 2, the heart segmentation model achieved 100% sensitivity and specificity.

**Table 2:** Heart identification by the model on the test set

	Heart signal detected by the model	No heart signal detected by the model
Heart signal by reference	103	0
No heart signal by reference	0	10

## Discussion

**Fig. 12:**
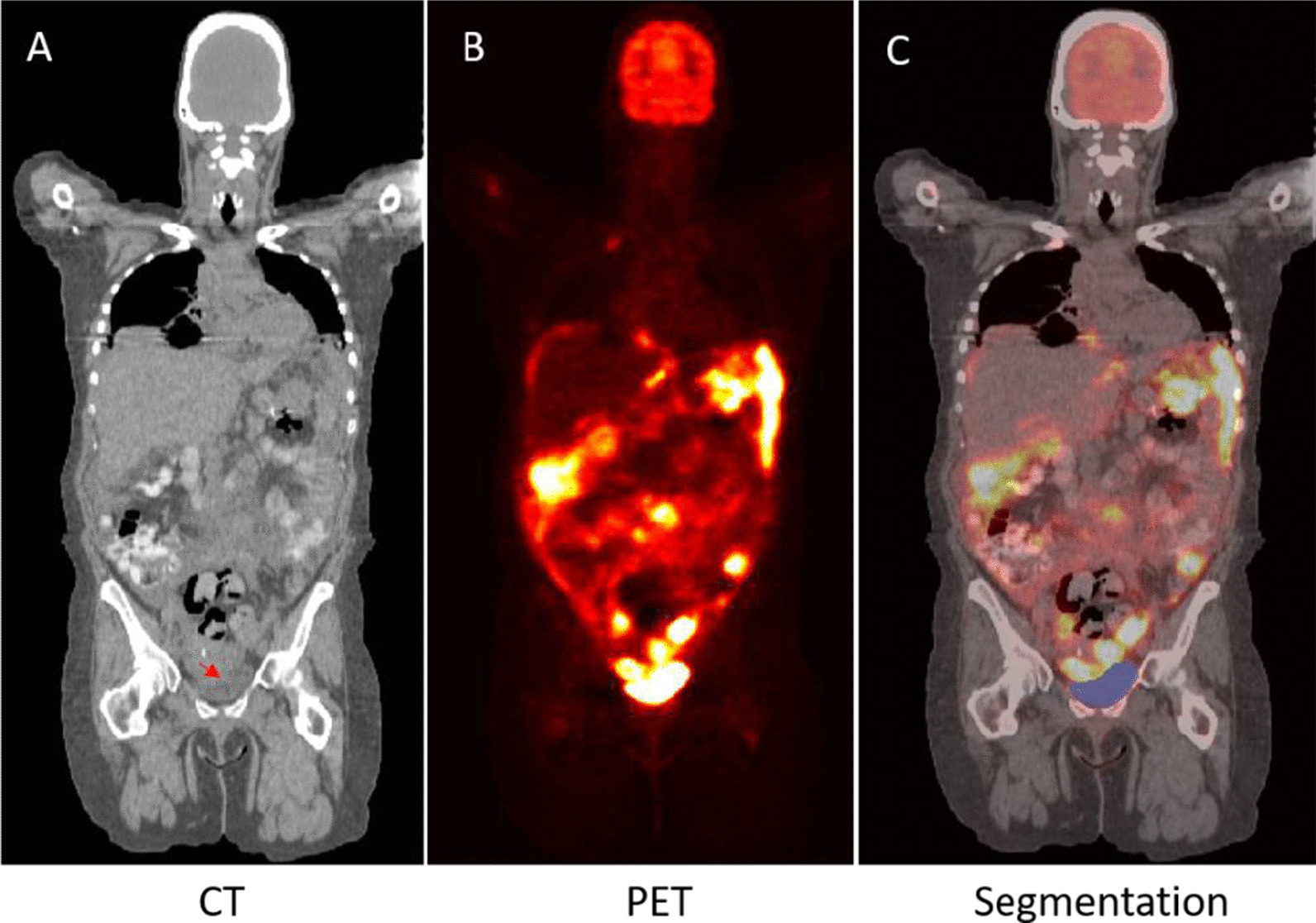
**a** CT image, **b** PET image, **c **segmentation by the model with mask overlaid in blue. The red arrow on CT image points out the bladder and tumor boundary

Physiological noise arising from FDG accumulation in the heart and bladder complicates the analysis of FDG-PET images. We develop two deep learning 3D models to perform heart and bladder segmentation in FDG PET/CT images to remove this physiological FDG accumulation within these organs to simplify any further downstream analysis. This is the first application of DL methodology to address the suppression of FDG signal coming from the bladder and heart. We constructed large training and test sets from two independent trials that were used in model development and evaluation. There is no additional post-processing applied to the model output. In this application, FDG-PET data is primarily providing the signal to identify high metabolic regions while the CT scan can assist with essential anatomical landmarks to localize the heart and bladder. As such, the model likely takes greater advantage of the FDG-PET signal when the heart or bladder appear different on the CT to ensure the segmentation completeness. On the other hand, CT likely plays a more significant role when a tumor is close or attached to the heart or bladder by providing greater anatomical information, and, thus, contributes to differentiating lesions from the surrounding physiological noise (normal bladder or heart uptake). This can be seen in Fig. 12, where no boundary exists between the organ and the lesion in the FDG image (Fig. 12b), and, thus the differentiation is likely driven by the boundary visible in the CT (Fig. 12a, red arrow) leading to the successful bladder segmentation shown in Fig. 12c.

The complementary multi-modal inputs to the model contribute to the model’s ability to resolve the under-investigated problem of FDG-PET false positives due to physiological noise in clinical practice. This methodology could be a valuable component of an imaging pre-processing step prior to tumor characterization by manual, semi- or automated tumor analysis methods.

The heart segmentation model does not segment the entire heart but was trained to only identify areas with high FDG uptake within the heart. Full heart segmentation is actually unnecessary in this setting because the ultimate goal is to remove the FDG signal from the heart to minimize false positives in FDG-based tumor identification. As such, this task may slightly differ from regular heart segmentation on CT [35, 36] or MR [37, 38], but, also requires both CT and FDG-PET data. The uptake of FDG in the heart muscle of patients undergoing chemo-therapy has been investigated [39] and focal myocardial FDG uptake indicates a significant increased risk for multiple myocardial abnormalities. As such, this heart segmentation model could be used as an automated tool to assist the measurement of myocardial uptake.

The heart model was found to perform very well in cases where there was no visible FDG heart uptake and did not classify any heart voxels as positive for FDG uptake for the negative (no visible heart uptake) patients. This was accomplished by including patients in the training data set that did not exhibit cardiac FDG uptake in their FDG PET images. We also attempted to include negative cases (no FDG bladder uptake) for the bladder segmentation model development. But, all patients in the training and test data sets exhibited some level of FDG signal in their bladder. This may be a limitation to this study since the bladder algorithm was not evaluated on patients void of FDG signal in their bladder. It will be interesting to assess the robustness of the model if data becomes available where the bladder is completely empty at the time of the scan. An additional limitation of this study is fact that patients were not required to empty their bladders before entering the scanner. This likely led to the high inter-patient variability that was observed in the size of the bladder. Another potential limitation of this study is that these models were only developed and tested on NHL patients. It is possible that algorithm performance could vary in diseases where lesion FDG avidity and location is different.

FDG PET assessment of TMTV has been found to be a prognostic biomarker that can be used to identify patients at high risk of early progression [6–8]. Traditional threshold based method could mistake heart or bladder as tumor and this could be especially problematic for patients with low tumor burden. As a result, these errors could lead to erroneous conclusions on the efficacy of drugs and cause unnecessary treatment burden on patients. The automatic elimination of physiological noise from heart and bladder by deep learning models will help to provide a more precise measurement of metabolic tumor burden and potentially lead to better management of patients. We will further investigate the robustness of the bladder and heart segmentation models in patients with other cancer types to improve the generalizability of the models.

## Conclusion

We present a deep learning approach to automatically segment heart and bladder on whole body FDG PET/CT, as a means to minimize potential false positives from being included in tumor identification. The model demonstrated rigorous performance regardless of varied location, size and shape of the target organ, and confusion of surrounding tumors.

## Data Availability

The datasets during and/or analyzed during the current study available from the corresponding author on reasonable request.
